# Translational metagenomics

**DOI:** 10.1186/s12967-021-02835-0

**Published:** 2021-04-19

**Authors:** Selvasankar Murugesan, Souhaila Al Khodor

**Affiliations:** Maternal and Child Health Program, Sidra Medicine, Doha, Qatar

We are pleased to announce the unveiling of a new section entitled “Translational Metagenomics” in the Journal of Translational Medicine. The core objective of this new section is to embrace studies addressing the role of the human microbiome in health and disease. In addition, this section encompasses studies about the development and application of metagenomics, metatranscriptomics, microbial metabolomics or novel bioinformatics tools used to improve our knowledge on the microbiome. Translational Metagenomics section is especially interested in studies that go beyond description of the microbial composition and include experimental models used to support the proposed role of the microbiome in human health and diseases. In addition studies about metagenomics integration with other omics are highly recommended. Studies of pathogens interaction with the host will not be considered.

The human body is home to a large number microbes that inhabit various internal and external surfaces. Recent breakthroughs in sequencing technologies combined with a better computational framework and bioinformatics tools have enabled researchers to understand the composition of the microbiome and how these microbes are involved in human health and disease. The microbiome and its mediators (such as microbial metabolites) are in a continuous cross talk with our immune system. Imbalance in the microbiome compostion refered to as dysbiosis was shown in many chronic and infectious diseases.

The use of omics technologies enabled researchers to assess the microbiome at various levels including composition, function, gene expression, protein expression and microbial metabolites among others. 16sRNA gene sequencing and shotgun metagenomics can be used to analyze human microbiome composition and diversity in samples collected from various body sites. Besides, functional metagenomics can identify novel functional genes, microbial pathways, antibiotic resistance genes and functional dysbiosis of the human microbiome. Other applications such as metatranscriptomics, metaproteomics, and metabolomics are also used to complement our understanding of the human microbiome.

A major concern in this field of research is the challenge in handling the samples and analyzing those large datasets. Technical variation can occur during different stages of the sample processing including collection method, sample handling and storage in addition to the protocol used for DNA extraction that can introduce biases towards certain microbial taxa. On the other hand, researchers face statistical and computational challenges related to data normalization and accurate quantification of the microbial taxa, relative gene abundance and their metabolic activities; precise phylogenetic placement of genomes in addition to the multivariate but sparse analysis of high-dimensional compositional data. It is vital to use adequate analytical pipelines to present this data to the scientific community in an easily interpretable model that is also reproducible.

The main goal of this section from the Journal of Translational Medicine is to fill the gap between basic research, translational medicine, and high-throughput data analysis for the human microbiome by providing innovative and novel scientific ideas. To ensure the best outreach to the scientific community, the journal offers a high standard peer review process and facilitates open access to high-quality research articles. The Editorial Panel is looking forward to receiving your manuscripts.

